# Supplementary material for: Exploring acculturative stress and coping mechanisms among pregnant South Asian immigrants in Ontario, Canada: A qualitative study protocol
Source: PLoS One. 2026 Mar 27;21(3):e0345952. doi: 10.1371/journal.pone.0345952 (PMC13028355; doi:10.1371/journal.pone.0345952)
Supplement: S1 File — (DOCX) [file pone.0345952.s001.docx]

### **Supporting File 1: Interview Guide**

**Project Title:** *Exploring Acculturative Stress and Coping Mechanisms Among Pregnant South Asian Immigrants in Ontario, Canada*
 **Target Participants:** Pregnant South Asian immigrants residing in Ontario
 **Interview Length:** Approximately 45–60 minutes
 **Interview Format:** Semi-structured, virtual

### **Opening Script (to be read by the interviewer):**

Thank you for taking the time to participate in this interview. We’re conducting a research study to better understand the experiences of pregnant South Asian immigrants in Ontario—particularly the kinds of stress you may experience while adjusting to life in a new country, and the ways you cope with these challenges.

Your perspective will help us and our partner organization, CASSA, improve services and support for pregnant immigrants. This interview will be audio-recorded, and your responses will be kept strictly confidential and used only for research purposes.

There are no right or wrong answers—please feel free to share whatever you are comfortable with.

*Do you have any questions before we begin?*

## **SECTION 1: Migration Context**

1. To begin, can you tell me a little about yourself?
   (Prompt: country of origin, year of arrival, family makeup, reason for migrating)
2. How did you decide to move to Canada, and what were your hopes or expectations?
3. Did you have any support system (friends, family, community) here when you arrived?
4. What have been the biggest changes or adjustments for you since moving to Ontario?
5. Looking back, how would you describe your first few months here?
   (Probes: challenges with housing, employment, language, or social connections)
6. How has your sense of “home” changed since you arrived?

## **SECTION 2: Pregnancy-specific Cultural Navigation**

1. How has your pregnancy journey been so far in Canada?
   (If applicable: How does this compare to previous experiences in your home country?)
2. Have you noticed any differences in how pregnancy is portrayed or discussed in public here versus in your culture? Has this affected how you dress, carry yourself, or feel about your body during pregnancy?
3. Have you experienced challenges navigating the healthcare system while pregnant?
   a. How did you manage those challenges?
   b. Were there people or organizations who helped you along the way?
4. Have there been any emotional or mental wellbeing challenges related to adapting during pregnancy? (Probes: loneliness, stress, joy, guilt, loss, sense of belonging)

## **SECTION 3: Acculturative Stress**

1. Overall, what has been the most stressful part of adjusting to a new culture while being pregnant?
2. Can you describe a moment when you felt emotionally or culturally out of place during your pregnancy?
   (Probes: religious norms, language, healthcare expectations, family roles)
3. Have you found it easy or difficult to follow your traditional diet or pregnancy food practices in Canada?
   1. *What challenges have you faced around finding or preparing familiar foods?*
   2. *Did healthcare advice about diet ever conflict with your cultural practices?*
4. Have you experienced any pressure to act or think differently to “fit in” in Canada?
5. Has being pregnant in a new country changed how you see yourself — as a South Asian woman, as a mother?
6. Have you encountered any discrimination, bias, or racism during your pregnancy journey?
   a. What impact did this have on your well-being or confidence?
7. What roles have language, gender norms, or unfamiliar systems (e.g., healthcare, insurance) played in shaping your experience?
   (Probes: has anything made you feel excluded or empowered?)
8. Have you ever felt a conflict between your family or cultural community’s expectations and the norms here in Canada during pregnancy?
   *(Probes: partner involvement, working during pregnancy, expectations around rest or decision-making)*
9. Have older family members (e.g., parents, in-laws) played a role in how you approach pregnancy here? Have their expectations ever conflicted with what’s available or expected in Canada?

## **SECTION 4: Coping Strategies and Social Support**

1. When you’ve felt stressed or overwhelmed, what has helped you get through it?
   (e.g., prayer, family support, community, cultural practices, therapy)
2. Have online platforms (social media groups) helped you stay connected to your cultural roots or made you feel more included in your pregnancy journey?
   1. Which ones?
   2. What kind of connection or help did you find there?
   3. Have they also made you feel more disconnected at times?
3. Have you found safe spaces (apart from online platforms) or people in Canada who make you feel understood and supported?
4. What personal strengths or inner resources have helped you cope during this time?
   (e.g., adaptability, faith, humor, previous life experiences)

## **SECTION 5: Recommendations**

1. Have you come across any services that felt culturally respectful or tailored to your needs?
   (e.g., language support, cultural food in hospitals, spiritual care)
2. What services or types of support do you think are missing or could be improved?
3. What recommendations would you give to healthcare providers, settlement workers, or community organizations to better support pregnant immigrant women?
4. What advice would you give to other South Asian women who are planning to migrate or are newly pregnant in Canada?

## **Closing Questions and Wrap-Up**

1. Is there anything we didn’t ask that you think is important for us to understand about your experience?
2. Would you be open to reviewing a short summary of our findings and sharing your feedback at a later stage?
   (This would take 20–30 minutes and would help ensure your voice is accurately reflected.)

Thank you again for your time and honesty. Your insights are incredibly valuable, and they will help shape more responsive and inclusive programs for immigrant women like yourself.
