# Supplementary material for: Exploring acculturative stress and coping mechanisms among pregnant South Asian immigrants in Ontario, Canada: A qualitative study protocol
Source: PLoS One. 2026 Mar 27;21(3):e0345952. doi: 10.1371/journal.pone.0345952 (PMC13028355; doi:10.1371/journal.pone.0345952)
Supplement: Attachment — Submitted filename: Point by point response-AF copy.docx [file pone.0345952.s003.docx]

Dear Ietza Bojorquez,

PLOS One

We are very thankful for editor’s comments and are pleased to submit the revised research manuscript titled “Exploring acculturative stress and coping mechanisms among pregnant South Asian immigrants in Ontario, Canada: A qualitative study protocol” (PONE-D-26-01077).

A point-by-point response is provided below in the table.

We look forward to a publication of our manuscript in PLOS One

Sincerely,

**Anam Shahil-Feroz** (she/her)

Assistant Professor

Arthur Labatt Family School of Nursing, Faculty of Health Sciences

FIMS & Nursing Building Room 3338

Western University, London ON Canada  N6A 5B9

<https://www.uwo.ca/fhs/nursing/about/faculty/research_supervisors/anam_s.html>

**EDITOR COMMENTS**

| **S. No.** | **Comment** | **Point by point response** |
| --- | --- | --- |
| 1. | In the Financial Disclosure, please specify if the funders played any role in the study design, data collection, analysis, decision to publish or preparation of the manuscript. | We have now clarified in the manuscript that the funders had no role in the study design, data collection, data analysis, decision to publish, or preparation of the manuscript. This statement has been explicitly added to the Funding section on Pg. 10. |
| 2. | According to the title, the aim is to explore acculturative stress and “coping mechanisms”, then in the aims “coping strategies” are mentioned. Are mechanisms and strategies the same? | We agree that the terminology should be consistent. In this context, “coping mechanisms” and “coping strategies” refer to the same concept. To ensure clarity and consistency throughout the manuscript, we have revised the text to use “coping mechanisms” consistently. |
| 3. | In the Introduction, acculturation is described as “a process where individuals strive to preserve their cultural identity while adapting to Canadian values and norms”. But acculturation doesn’t refer exclusively to Canadian values. Please provide a more general description of this construct. | Thank you for this important comment. We agree that acculturation is not specific to Canadian values but refers more broadly to the process of cultural and psychological change that occurs when individuals come into contact with a new cultural context. We have revised the Introduction to provide a more general definition of acculturation, removing the exclusive reference to Canadian values and norms. |
| 4. | The interviews will be conducted over zoom. What was the rationale for choosing this over in-person interviewing? Could you discuss the limitations that this form of interviewing implies? | Thank you for this important comment. We have now added a justification for conducting interviews via Zoom in the Methods section (Pg. 7). Specifically, we clarify that the decision was guided by considerations of accessibility, flexibility, and participant comfort, particularly given potential caregiving responsibilities, transportation barriers, and geographic dispersion among participants.  We have also included the limitations of virtual interviewing (Pg. 9), including potential challenges in establishing rapport, reduced observation of non-verbal cues, technological barriers (e.g., limited internet access or digital literacy), and privacy concerns in shared living environments. Additionally, we describe mitigation strategies, including providing technical assistance and referrals to organizations that offer access to digital devices and internet connectivity (e.g., libraries and community partners such as CASSA). |
| 5. | Please specify the currency of the honorarium to be given to participants. | Thank you for this comment. We have clarified on page 7 of the revised manuscript that participants will receive an honorarium of 20 CAD. |
| 6. | In the Data Analysis section, the reference for thematic analysis is Braun & Clark. But those authors’ reflexive approach is different from the interpretive description proposed by Thorne. Please specify which “flavor” of thematic analysis will be used for this project. | We thank the reviewer for this important point. We have clarified that the project employs a constructivist qualitative design using Braun & Clarke’s reflexive thematic analysis. The Study Design and Data Analysis sections have been revised to describe the iterative, reflexive, and interpretive coding process, emphasizing that themes are actively constructed by the researchers rather than passively discovered. We have also highlighted how reflexivity will be incorporated through researcher reflection, journaling, and analytic discussions. These revisions ensure methodological coherence and clearly specify the approach to thematic analysis used in this study. |
